# Impact of magnetic fields from tablets, laptops, smartphones, and household/leisure magnets on cardiac implantable electronic devices

**DOI:** 10.1002/joa3.70106

**Published:** 2025-06-30

**Authors:** Norio Kamitani, Aya Miyazaki, Satoko Tomida, Keita Shimizu, Nodoka Ohira, Keisyun Kondo, Hiromichi Miura, Daishi Koyama, Shigehiko Tominaga, Ryuta Henmi, Ryo Sugiura, Hiroshi Masui

**Affiliations:** ^1^ Department of Clinical Engineering Seirei Hamamatsu General Hospital Hamamatsu Japan; ^2^ Department of Pediatric Cardiology Seirei Hamamatsu General Hospital Hamamatsu Japan; ^3^ Department of Adult Congenital Heart Disease Seirei Hamamatsu General Hospital Hamamatsu Japan; ^4^ Department of Cardiology Seirei Hamamatsu General Hospital Hamamatsu Japan

**Keywords:** cardiac implantable electronic device, magnet field, magnet mode, magnetic flux density, tablet computer

## Abstract

**Background:**

Cardiac implantable electronic devices (CIEDs) activate the magnet response at a magnetic flux density of ≥10 gauss (G), which may cause unintended pacing, leading to discomfort or even severe arrhythmias. Information processing devices have recently incorporated magnets, which may activate the magnet mode in patients with abdominally implanted devices, subcutaneous implantable cardioverter‐defibrillators (ICDs), or extravascular ICDs.

**Methods:**

We investigated the effects of the magnetic fields generated by information processing devices (tablets, laptops, and smartphones) and household/leisure magnets on 13 models of CIEDs, analyzing their association with magnet mode activation in different manufacturers' CIEDs.

**Results:**

The tested magnet materials exhibited a maximum magnetic flux density of 290–1360 G. The magnetic flux density distribution in the information processing devices was as follows: accessory connectors, speakers, cameras, and microphones (*p* = 0.0001). The median activation distances for the magnet mode were 6.5 (range, 4–15), 5 (4–11.3), and 0.01 (activated only when attached; 0–7) mm for tablets and laptops, smartphones, and household/leisure magnets, respectively (*p* < 0.0001). The maximum distance at which the magnetic flux density decreased below 10 G was the longest for tablets and laptop computers at 18 mm.

**Conclusion:**

Information processing devices and household/leisure magnets can affect CIEDs when placed in close proximity. Among the devices tested, magnet mode activation did not occur at distances of ≥20 mm. Considering the increasing prevalence of information processing devices and the growing adoption of nonthoracic CIED placements, raising awareness among patients about potential interactions is crucial.

## Graphical abstract

This study on 13 Cardiac implantable electronic device (CIED) models revealed that surface magnetic fields of tablets, laptops, smartphones, and household magnets exceeded 10 G, potentially triggering magnet response mode. Activation occurred at ≤15 mm for electronic devices and ≤7 mm for household magnets. Magnet response was not observed >18 mm. These findings underscore the risk of unintended magnet response at close proximity, necessitating patient education and healthcare professional awareness regarding safe device use and variable magnet mode operation across CIED manufacturers.

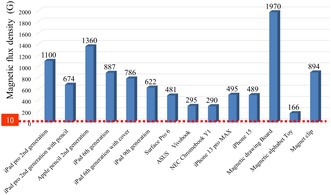

## 1 INTRODUCTION

Cardiac implantable electronic devices (CIEDs) have advanced from pacemakers to implantable cardioverter‐defibrillators (ICDs) and cardiac resynchronization therapy (CRT) devices, with the number of patients indicated for CIEDs annually increasing.^1^ Concurrently, the widespread adoption of several radio‐frequency devices has raised concerns regarding electromagnetic interference (EMI) with CIEDs. Specifically, sporadic reports of EMI caused by tablet devices^2^, ^3^, ^4^ and static magnetic fields from smartphones^5^, ^6^ have been conducted, increasing considerable concerns about inadvertent magnet mode activation.

In the magnet mode, pacemakers perform fixed‐rate pacing, disregarding intrinsic cardiac activity. This functionality was designed to avoid inappropriate pacing during procedures exposed to EMI. In ICDs, pacing functionality remains unchanged, while tachycardia detection is inhibited, anticipating the need for a temporary tachycardia therapy suspension.^5^ However, in pacemakers, the magnet mode can compete with the patient's natural heart rhythm, causing discomfort and potentially inducing arrhythmias through the Spike‐on‐T phenomenon. In ICDs, a risk exists that appropriate therapy may be withheld when a severe arrhythmia occurs during magnet mode activation. Therefore, for medical safety, preventing unintentional magnet mode activation in patients with CIEDs is essential.

The proximity to the generator during tablet or laptop use increases when the CIED generator is placed on the abdomen of patients with congenital heart disease or in pediatric patients or when newer devices, including subcutaneous ICDs (S‐ICDs) and extravascular ICDs (EV‐ICDs) are used, necessitating caution regarding magnet mode activation.^2^, ^7^ The minimum magnetic flux density reported to activate the magnet response in CIEDs is 10 gauss (G)^8^; however, to our knowledge, detailed studies on the effects of common magnetic materials on CIEDs are lacking. Therefore, this study aimed to investigate the influence of magnetic fields from commonly used information processing devices, including tablets, laptops, and smartphones, as well as household/leisure magnets, and their association with the magnet response.

## 2 METHODS

### 2.1 CIEDs and magnetic materials

The CIEDs used in this study comprised pacemakers, ICDs, and CRT devices (Table 1), with a total of 13 different models. Tablets, laptops, smartphones, and household/leisure magnets were the magnetic materials assessed (Table 2).

**TABLE 1:** Cardiac implantable electrical devices for evaluation.

Model	Manufacturer
Pacemaker	
Azure XT DR MRI	Medtronic plc
Accolade MRI DR EL	Boston Scientific Corporation
Amvia Sky DR‐T	Biotronik SE & Co. KG
Assurity MRI DR	Abbott Laboratories
Alizea SR	MicroPort Scientific Corporation
ICD	
Cobalt XT VR MRI SureScan	Medtronic plc
Momentum EL DR	Boston Scientific Corporation
Gallant DR	Abbott Laboratories
Platinum VR	MicroPort Scientific Corporation
CRT‐D	
Cobalt HF CRT‐D MRI SureScan	Medtronic plc
Momentum CRT‐D	Boston Scientific Corporation
Rivacor7 HF‐T	Biotronik SE & Co. KG
Gallant HF CRT‐D	Abbott Laboratories

**TABLE 2:** Magnet materials for evaluation.

	Manufacturer	Types of magnets
Tablet computer		
iPad pro 2nd generation	Apple Inc.	Neodymium
iPad pro 2nd generation with pencil	Apple Inc.	Neodymium
Apple pencil 2nd generation	Apple Inc.	Neodymium
iPad 6th generation	Apple Inc.	Neodymium
iPad 6th generation with cover	Apple Inc.	Neodymium
iPad 9th generation	Apple Inc.	Neodymium
Surface Pro 6	Microsoft Corporation	Neodymium
Laptop computer		
ASUS VivoBook	ASUSTeK Computer Inc.	Neodymium
NEC Chromebook Y1	NEC Corporation	Neodymium
Smartphone		
iPhone 13 pro MAX	Apple Inc.	Neodymium
iPhone 15	Apple Inc.	Neodymium
Magnetic drawing Board	Bell Ganka Co. Ltd.	Ferrite
Magnetic alphabet Toy	Kuno & Company Limited	Ferrite
Magnet clip	Sunnote Co., Ltd.	Ferrite

### 2.2 Magnetic flux density measurement

The magnetic flux density was measured using a gaussmeter (GM‐301, Electronic & Magnetic Industries Co., Ltd.). The instrument's measurement range was 0–3000 mT (0–30 000 G) in the DC mode and 0–1500 mT (0–15 000 G) in the AC mode. The measurement accuracies were specified as ± (2% + 0.3% F.S. + 4 dig) and ± (4% + 0.6% F.S. + 10 dig) for DC and AC measurements, respectively.

**FIGURE 1:**
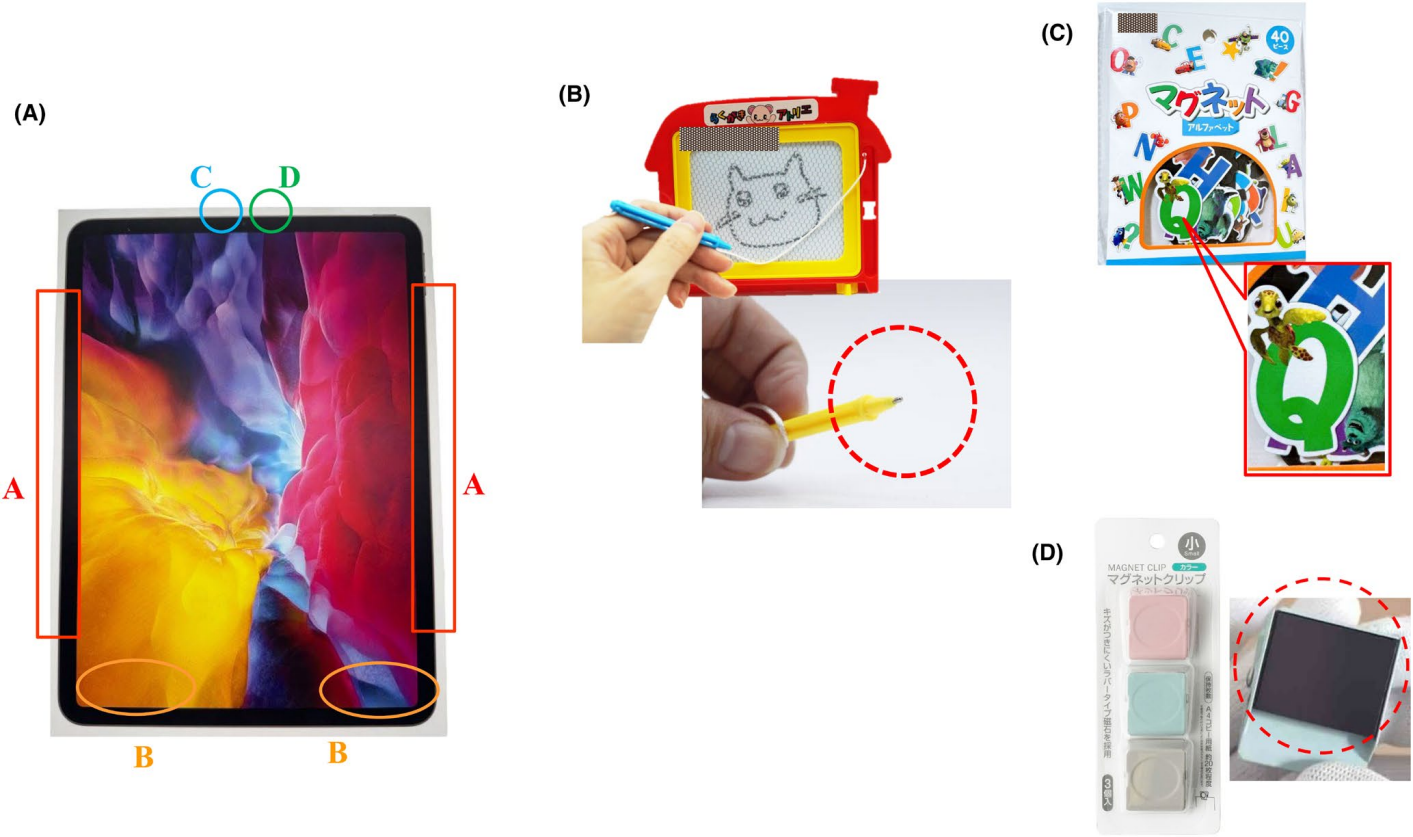
Parts of the evaluation of the magnetic field in the tablet, laptop, or smartphone (A); magnetic drawing board (B); magnetic alphabet toy (C); and magnetic clip (D). Figure (A) shows the iPad ninth generation. The red square, yellow circle, blue circle, and green circle indicate the accessory connection part, speaker, camera, and microphone, respectively.

First, under direct contact conditions, the magnetic flux density of each magnetic material was measured. For tablets, laptops, and smartphones, EMI was measured at the accessory connectors, speakers, cameras, and microphones, as these are common locations for integrated magnets (Figure 1A). Additionally, magnetic flux density measurements were performed on an iPad sixth generation with a smart cover attached and an iPad Pro second generation with an attached Apple Pencil. For household/leisure magnets, measurements were conducted at specific locations shown in Figure 1B–D.

Second, using the CIEDs, the activation distance for magnet mode was determined by measuring from the point of maximum magnetic flux density of each magnetic material. When the magnet mode was activated, only through direct attachment, the distance was set to 0.01 mm. When inactivated, even through direct attachment, the distance was set to 0 mm.

**FIGURE 2:**
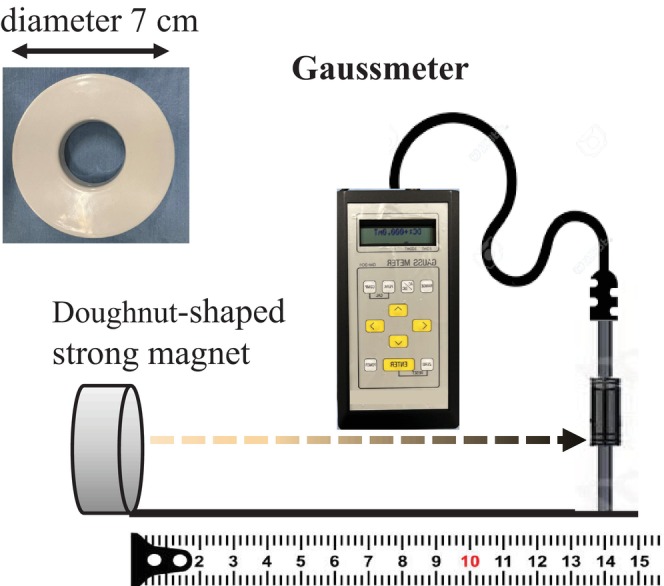
Measurement method for the association between the magnetic flux density and the distance in a doughnut‐shaped ferrite magnet.

Third, to describe the association between the magnetic flux density and the distance, a doughnut‐shaped ferrite magnet (diameter, 7 cm; surface magnetic flux density, 1000 G) was employed, and the magnetic flux density was measured at various distances from the magnet (Figure 2). An approximate formula for this association was derived using Microsoft® Excel® 2019 MSO (Version 2503, Build 16.0.18623.20208), 32 bit. With the magnetic flux density measurement range set at 1–100 G, the association between the magnetic flux density and the distance was measured at the location of the maximum magnetic flux density of the magnetic material, and the maximum distance at which the magnetic flux density decreased below 10 G was determined.

Fourth, the effect of speaker volume on the magnetic flux density was evaluated by varying the speaker volume in two models of tablet (Surface Pro 6) and laptop (VivoBook) and measuring the resulting changes in the magnetic flux density.

### 2.3 Statistical analysis

Values were presented as medians (in ranges). The Kruskal–Wallis test was used for unpaired comparisons of magnetic flux density among locations for integrated magnets in information processing devices as well as activation distances for magnet modes among the magnet materials and CIED categories. All statistical analyses were performed using JMP 18 statistical software (SAS Institute, Cary, NC, USA). A *p*‐value of <0.05 was considered statistically significant.

## 3 RESULTS

### 3.1 Magnetic flux density under contact conditions for each magnetic material

**FIGURE 3:**
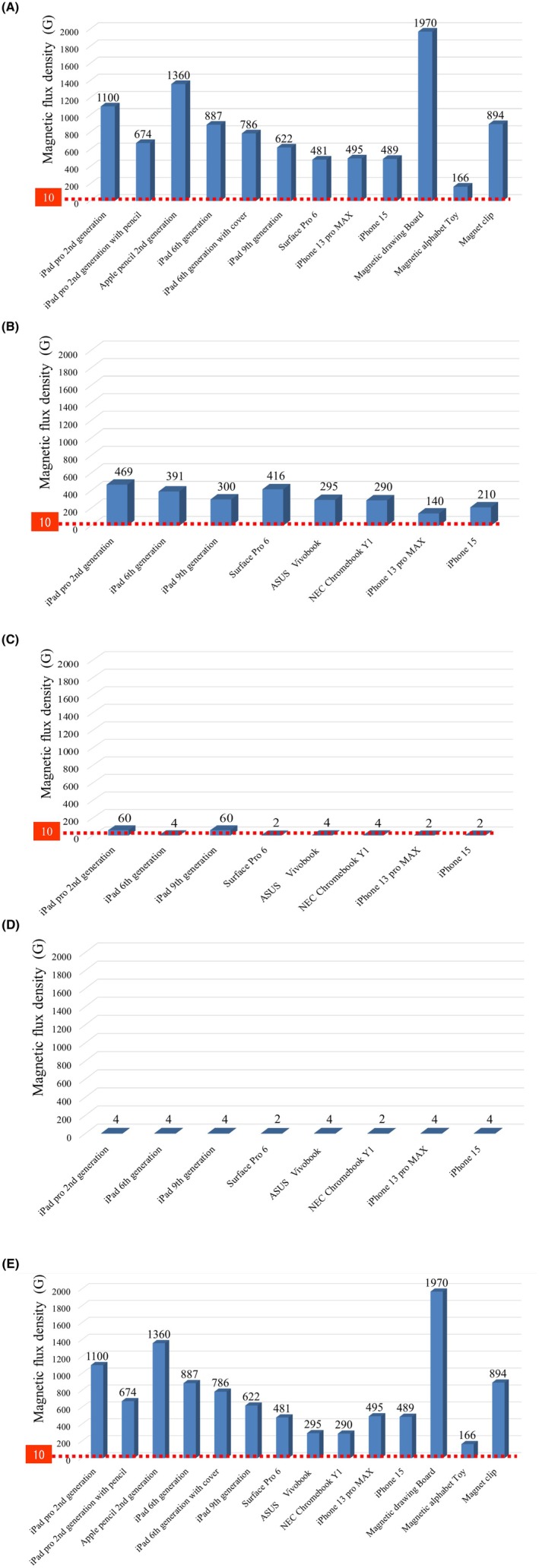
Magnetic flux density in each product. Magnetic flux densities at the accessory connection part, speaker, camera, and microphone of the tablet and laptop are presented in (A), (B), (C), and (D), respectively. (E) The maximum flux density in each product is shown. Detailed data are presented in Table S1.

The following was the magnetic flux density distribution (in G) for tablets, laptops, and smartphones in descending order: accessory connectors, 648 (481–1100), speakers, 297.5 (140–469), cameras, 4 (2–60), microphones, 4 (2–4) (*p* = 0.0001) (Figure 3A–D, Table S1). Household/leisure magnets demonstrated a wide range of magnetic flux densities, spanning from 135 to 1970 G (Figure 3A–D, Table S1). Figure 3E illustrates the maximum magnetic flux density for each magnetic material, with the magnetic drawing board demonstrating the highest value.

### 3.2 Activation distances for magnet mode in CIEDs

For pacemakers, the activation distances for the magnet mode were 6.5 (4–15), 5 (4–8), and 0.01 (activated only when attached, 0–6.5) mm for tablets and laptops, smartphones, and household/leisure magnets, respectively (*p* < 0.0001).

For ICDs, the activation distances for the magnet mode were 6.2 (5–12), 4.75 (5–12), and 0.01 (activated only when attached, 0–7) mm for tablets and laptops, smartphones, and household/leisure magnets, respectively (*p* < 0.0001).

For CRT defibrillators (CRT‐Ds), the activation distances were 6.5 (5–12), 5.05 (4–8), and 0.01 (activated only when attached, 0–6.1) mm for tablets and laptops, smartphones, and household/leisure magnets, respectively (*p* = 0.0005).

When all CIEDs were included, the activation distances for the magnet mode were 6.5 (4–15), 5 (4–11.3), and 0.01 (activated only when attached, 0–7) mm for tablets and laptops, smartphones, and household/leisure magnets, respectively (*p* < 0.0001) (Table 3).

**TABLE 3:** The activation distances for magnet mode (mm).

	Pacemaker					ICD				CRT‐D			
	Azure XT DR MRI	Accolade MRI DR EL	Amvia sky DR‐T	Assurity MRI DR	Alizea SR	Cobalt XT VR MRI SureScan	Momentum EL DR	Gallant DR	Platinum VR	Cobalt HF CRT‐D MRI SureScan	Momentum CRT‐D	Rivacor7 HF‐T^c^	Gallant HF CRT‐D
	Medtronic	Boston Scientific	Biotronik	Abbott	MicroPort	Medtronic	Boston Scientific	Abbott	MicroPort	Medtronic	Boston Scientific	Biotronik	Abbott
Tablet computer													
iPad pro 2nd generation	15	5	7	6.5	5	6.5	10	6	5	6.5	12	N/A	6.5
iPad 6th generation	13	5	8	7.5	6	6	12	6.4	6	7	12	N/A	7
iPad 9th generation	8	4.5	4	6.5	5	5	5	6.5	5	5	5.5	N/A	6.5
Surface Pro 6	12	7	9	5	7	5	8	7	7	5	8	N/A	6
Laptop computer												N/A	
ASUS VivoBook	13	6	10	6.5	6	5	8.5	10.5	6	5	7	N/A	10
Smartphone												N/A	
iPhone 13 pro MAX	8	5	5	8	4.5	5.5	4	11.3	4.5	5	4	N/A	8
iPhone 15	6.5	5	4	6	4	6.4	4	5	4	5.1	4	N/A	6.5
Magnetic Drawing Board	1	0.01^a^	0.01^a^	0.01^a^	0.01^a^	0.01^a^	0.01^a^	0.01^a^	0.01^a^	0.01^a^	2	N/A	0.01^a^
Magnetic Alphabet Toy	0^b^	0^b^	0^b^	0^b^	0^b^	0^b^	0^b^	0^b^	0^b^	0^b^	0^b^	N/A	0^b^
Magnet Clip	4	4	3	6.5	6.5	3.6	5	7	4	4.2	5	N/A	6.1

Abbreviations: CRT‐D, Cardiac Resynchronization therapy Defibrillator; ICD, implantable cardioverter‐defibrillators; N/A, not applicable.

^a^ Activated only when attached.

^b^ Not activated even when attached.

^c^ Biotronik CRT‐D systems do not exhibit any functional response to magnets.

The activation distances among the CIED categories were not statistically different for tablet/laptops, smartphones, and household/leisure magnets.

### 3.3 Association between the magnetic flux density and distance for each magnetic material

**FIGURE 4:**
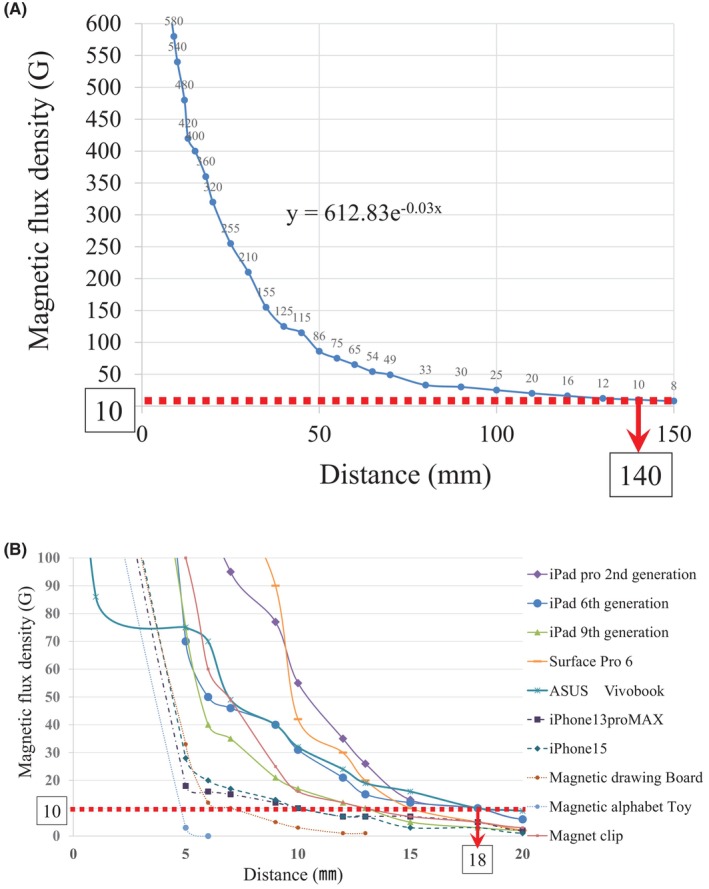
Magnetic flux density as a function of the distance from each magnetic material. (A) Doughnut‐shaped magnet and (B) magnetic materials.

For the doughnut‐shaped magnet, the magnetic flux density decreased as the distance from the magnet increased. This relationship was modeled by the following exponential decay equation: *y* = 612.83e−0.03*x* (Figure 4A).

Regarding the association between the magnetic flux density and the distance for the tested devices, the maximum distance at which the magnetic flux density decreased below 10 G was 18 mm for the iPad sixth generation, iPad Pro second generation, and VivoBook (Figure 4B).

### 3.4 Effect of speaker volume on the magnetic flux density

Varying the speaker volume from the minimum to the maximum in tablets and laptops did not produce any discernible changes in the magnetic flux density for either device. For the tablet (Surface Pro 6), the magnetic flux density was 416 G with the speaker off and remained at 416 G at the maximum volume. For the laptop (VivoBook), the magnetic flux density was 295 G with the speaker off and remained at 295 G at the maximum volume.

## 4 DISCUSSION

The magnetic flux density measurements showed that the surface magnetic fields of tablets, laptops, smartphones, and household/leisure magnets significantly exceeded 10 G, a level that can trigger the magnet mode in CIEDs. Considering that tablets and accessories, such as pencils, integrate magnets, it was hypothesized that the magnetic flux density would increase upon connection. Notably, despite the robust retention force between these devices, the magnetic fields only partially overlapped, yielding a weaker resultant force and no observed increase in the magnetic flux density.

CIEDs are programmed to switch to the magnet mode when an internal magnetic field detection component, including a reed switch or Hall sensor, is exposed to a static magnetic field exceeding a particular threshold.^9^, ^10^ In this study, the activation distances for the magnet mode were ≤15, ≤11.3, and ≤7 mm for tablets and laptops, smartphones, and household/leisure magnets, respectively (*p* < 0.0001). The effect of the magnetic flux density did not substantially differ between the device categories. Although minor variations of a few millimeters may occur owing to differences in the location of the magnetic field detection component within individual CIEDs, a separation distance of ≥20 mm from the CIED body is considered to provide a safe margin.

The magnetic flux density from the tested devices decreases as the distance increases, in inverse proportion to the square of the distance.^11^ Similarly, the present study exhibited this inverse square relationship. Moreover, the attenuation of magnetic flux density significantly varies depending on the size and shape of the magnet. In this study, the attenuation observed with a 7‐cm‐diameter doughnut‐shaped magnet substantially differed from that of small magnets noted in other materials. Larger magnets tend to have a wider magnetic field and exhibit more gradual attenuation over distance, whereas smaller magnets demonstrate significantly steeper attenuation. Therefore, even with similar surface flux densities, small magnets may affect CIEDs only at close ranges. These findings suggest that the size and shape of the magnet should also be considered when assessing EMI risks.

Magnets are categorized into the following four types on the basis of their constituent materials: neodymium magnets, ferrite magnets, samarium–cobalt magnets, and Alnico magnets. Neodymium and ferrite magnets are frequently used in peripheral devices. Neodymium magnets, characterized by their high magnetic flux density despite their small size, are predominantly used in precision equipment, such as terminal devices. Ferrite magnets, which can be manufactured in various shapes, are broadly employed in household/leisure products. In this study, compared with tablets, laptops, and smartphones, household/leisure magnets (ferrite magnets) demonstrated the highest magnetic flux density when directly measured at the magnet surface. However, the separation distance required for preventing the CIED interaction was relatively short, suggesting that the differences in the magnetic influence can be associated with the magnet material.

The Ministry of Internal Affairs and Communications of Japan, in its “Guidelines for preventing the effects of radio waves from various radio‐controlled devices on implantable medical devices,” recommends maintaining a minimum of 15‐cm distance between mobile phone terminals and the implanted medical device.^12^ This recommendation is based on the electromagnetic compatibility requirements in ISO 14117 as well as the observation that an 800‐MHz band W‐CDMA (Wideband Code Division Multiple Access) mobile phone caused a level 2 interference with an implantable cardiac pacemaker at a 3‐cm distance. Conversely, the current model of 4 and 5G mobile phones revealed no such interference.^12^ Similar to radio waves, maintaining an adequate distance (15 cm) can mitigate the effects of magnetic fields. However, in the future, recommendations regarding separation distance should more clearly differentiate between the risks posed by radio waves and those posed by static magnetic fields.

Regarding the effect of speaker volume on the magnetic flux density in tablets and laptops, the speaker unit comprises a coil (voice coil) and a permanent magnet. When an electrical signal is applied to the coil, it moves back and forth according to Fleming's left‐hand rule; this motion is transmitted to the diaphragm, thereby generating sound. The greater the current and magnetic flux density, the greater the force with which the diaphragm can vibrate, causing increased sound output. Consequently, the magnetic flux density within a fluctuating magnetic field may change with variations in the volume. However, as this study focused on the effect of the static magnetic field generated by the magnet, no changes were observed in the maximum magnetic flux density for either device when the speaker volume was altered.

Although magnet mode activation from devices, including tablets, laptops, smartphones, and household/leisure magnets, is infrequent in adults (who typically have CIEDs implanted in the chest region), caution is warranted in several patient populations, including patients with abdominal CIED implantation; those with congenital heart disease; pediatric patients; and those with newer generation devices, including S‐ICDs and EV‐ICDs. A nationwide questionnaire survey conducted across 119 institutions encompassing patients with abdominal CIEDs reported a total of 2411 devices. Among these patients, magnet response events occurred in 31 cases, with the incidents taking place at schools (35.5%), at home (9.7%), and in unknown settings (54.8%).^13^ Among infants, playing with magnetic toys that come into contact with the abdominal CIED without appropriate caution may trigger magnet response. Among school‐aged children, tablets are frequently used for educational purposes, and using the device in close proximity to the abdomen may inadvertently activate the magnet mode.^2^ Even in adults, the tablet may be positioned near the abdominal CIED when patients lie down or on their feet when sitting cross‐legged. Regarding S‐ICDs, the number of implantations has increased since their approval in Japan in 2016, reaching around 800 cases in 2022, particularly among relatively young and active patients.^14^ In 2025, EV‐ICDs will become available in Japan, and the number of implantations is anticipated to increase. When these individuals carry tablets or laptops under their arms, a common behavior, the devices can be positioned near the S‐ICD or EC‐ICD, potentially leading to unintended magnet mode activation.

Educating patients regarding the significance of maintaining a safe separation distance from these devices is crucial.^15^ Furthermore, medical professionals should recognize that the operating behavior in the magnet mode varies by manufacturer and model. Additionally, certain models from some manufacturers do not beep or generate log records when the magnet mode is activated, making detection more difficult.^15^

## 5 LIMITATIONS

This study was an experimental investigation and did not replicate the anatomical and physiological conditions of the human body. Clinically, CIEDs are usually implanted beneath multiple layers of skin, subcutaneous fat, muscle, and vascular tissue, all of which may, to some extent, attenuate external magnetic fields. Therefore, the activation distances for the magnet mode observed in this study may overestimate the actual risks encountered in clinical settings. Furthermore, this study was conducted using readily available magnet materials and CIEDs provided through the cooperation of manufacturers; therefore, it did not comprehensively cover all types of magnet materials and CIEDs currently in use. Nevertheless, this study offers valuable insights into the potential effects of magnetic fields generated by materials frequently encountered in everyday life on CIEDs.

## 6 CONCLUSION

This study suggests that tablets, laptops, smartphones, and household/leisure magnets can trigger the magnet mode in CIEDs when in close proximity. In the evaluated devices, magnet mode activation was not observed at distances >18 mm. However, the use or transport of these devices at distances <18 mm from a CIED represents a potential risk of an unintended magnet response. Therefore, enhancing awareness of these risks, promoting safety consciousness among individuals with CIEDs, and providing appropriate guidance regarding the safe use of these electronic devices are significant.

## AUTHOR CONTRIBUTIONS

All authors substantially contributed to the conception and design of the study; the preparation of materials; data collection, analysis, and interpretation; or the drafting and revision of the manuscript. All authors read and approved the final manuscript and agreed to be accountable for all aspects of the study.

## CONFLICT OF INTEREST STATEMENT

Authors declare no conflict of interests for this article.

## APPROVAL OF THE RESEARCH PROTOCOL

No human participant was involved in this study.

## Supporting information


Table S1.

## Data Availability

The data supporting the findings of this study are available in the main text or in the supplementary materials of this article.
