# Supplementary material for: Impact of magnetic fields from tablets, laptops, smartphones, and household/leisure magnets on cardiac implantable electronic devices
Source: J Arrhythm. 2025 Jun 30;41(4):e70106. doi: 10.1002/joa3.70106 (PMC12209319; doi:10.1002/joa3.70106)
Supplement: Supplementary file 1 — Table S1. [file JOA3-41-e70106-s001.docx]

Supplementary table 1. Magnet flux density (G) in each magnet material

|  | Tablet computer | | | | | | |  | Laptop computer | |  | Smartphone | |  | Magnetic  drawing board | Magnetic alphabet toy | Magnet clip |
| --- | --- | --- | --- | --- | --- | --- | --- | --- | --- | --- | --- | --- | --- | --- | --- | --- | --- |
|  | iPad Pro  2nd generation | iPad Pro  2nd generation with pencil | Apple Pencil  2nd generation | iPad 6th generation | iPad 6th generation with cover | iPad 9th generation | Surface Pro 6 |  | ASUS  Vivobook | NEC Chromebook Y1 |  | iPhone 13 Pro Max | iPhone 15 |  |  |  |  |
| Accessory connection part | 1100 | 674 | N/A | 887 | 786 | 622 | 481 |  | N/A | N/A |  | 495 | 489 |  | N/A | N/A | N/A |
| Speaker | 469 | N/A | N/A | 391 | N/A | 300 | 416 |  | 295 | 290 |  | 140 | 210 |  | N/A | N/A | N/A |
| Camera | 60 | N/A | N/A | 4 | N/A | 60 | 2 |  | 4 | 4 |  | 2 | 2 |  | N/A | N/A | N/A |
| Microphone | 4 | N/A | N/A | 4 | N/A | 4 | 2 |  | 4 | 2 |  | 4 | 4 |  | N/A | N/A | N/A |
| Maximum | 1100 | 674 | 1360 | 887 | 786 | 622 | 481 |  | 295 | 290 |  | 495 | 489 |  | 1970 | 166 | 894 |

Abbreviations: N/A, Not applicable
